# Comparing Bioelectrical Impedance Analysis, Air Displacement Plethysmography, and Dual-Energy X-Ray Absorptiometry for Body Composition in Pediatric Obesity

**DOI:** 10.3390/nu17060971

**Published:** 2025-03-10

**Authors:** Alexandra Thajer, Martin Vasek, Sophie Schneider, Alexandra Kautzky-Willer, Franz Kainberger, Sebastian Durstberger, Andreas Kranzl, Brian Horsak, Susanne Greber-Platzer

**Affiliations:** 1 Division of Pediatric Pulmonology, Allergology and Endocrinology, Department of Pediatrics and Adolescent Medicine, Medical University of Vienna, 1090 Vienna, Austria; martin.vasek@meduniwien.ac.at (M.V.); sophies.e-mail@web.de (S.S.); susanne.greber-platzer@meduniwien.ac.at (S.G.-P.); 2 Gender Medicine Unit, Division of Endocrinology and Metabolism, Department of Internal Medicine III, Medical University of Vienna, 1090 Vienna, Austria; alexandra.kautzky-willer@meduniwien.ac.at; 3 Division of Neuro- and Musculoskeletal Radiology, Department of Biomedical Imaging and Image-Guided Therapy, Medical University of Vienna, 1090 Vienna, Austria; franz.kainberger@meduniwien.ac.at; 4 Department Health Sciences, FH Campus Wien—University of Applied Sciences, 1100 Vienna, Austria; sebastian.durstberger@fh-campuswien.ac.at; 5 Orthopaedic Hospital Speising, Laboratory of Gait and Movement Analysis, 1130 Vienna, Austria; andreas.kranzl@oss.at; 6 Institute of Health Sciences, St. Pölten University of Applied Sciences, 3100 St. Pölten, Austria; brian.horsak@fhstp.ac.at; 7 Center for Digital Health & Social Innovation, St. Pölten University of Applied Sciences, 3100 St. Pölten, Austria

**Keywords:** bioelectrical impedance analysis, air displacement plethysmography, dual-energy X-ray absorptiometry, body composition, obesity, children, adolescents

## Abstract

Background: Body composition analysis is crucial in clinical practice, yet few methods are suitable for pediatric patients, and data on young children with obesity are limited. Methods: This study measured body fat percentage (BFP), fat mass (FM), and fat-free mass (FFM) in 26 pediatric patients with obesity (BMI: 35.6±6.9 kg/m^2^), using two bioelectrical impedance analysis (BIA) devices (TANITA and BIACORPUS), and the results were compared to those of the gold-standard dual-energy X-ray absorptiometry (DXA). Additionally, air displacement plethysmography (BODPOD) was compared with DXA, and all methods were evaluated against each other. Results: Significant differences were observed between all methods and parameters (*p* < 0.05). For example, Bland–Altman analysis for BFP identified differences between BIACORPUS and DXA (mean: −3.5%; 95% limits of agreement: −16.7% to 9.8%) and between TANITA and DXA (mean: −3.1%; 95% limits of agreement: −12.2% to 6.1%). These differences can be regarded as clinically relevant, especially when considering the 95% limits of agreement. Further, moderate differences between BODPOD and DXA were identified, which could be clinically relevant (mean: 2.1%; 95% limits of agreement: −4.2% to 8.5%). Conclusions: TANITA was the most comparable BIA method to the gold standard, DXA. Therefore, TANITA is recommended for assessing body composition in young patients with obesity to monitor therapy progress in clinical settings. When using BODPOD as an alternative to DXA, caution is warranted since we found relevant differences between both methods.

## 1. Introduction

Body composition analysis can provide an overview of an individual’s nutrient supply by revealing acute and chronic forms of malnutrition, as well as overnutrition [1,2]. The most accurate information about the state of health is obtained when as many body composition factors as possible are known [3]. One important factor for assessing individual health and nutritional status is body fat [4]. The main characteristic of obesity is the excess of body fat. The body mass index (BMI) is a fast and inexpensive tool, but it has its limitations and might overestimate or underestimate obesity [5]. A clinical assessment of obesity has announced that, in addition to BMI, a minimum of one anthropometric measure (e.g., waist circumference and waist-to-height ratio) has to be used, or the direct determination of body fat by body composition analysis (e.g., DXA or BIA) can confirm the excess adiposity [6]. Body composition analysis helps to correctly assess obesity and to determine the risk of developing comorbidities. In addition, when assessing body fat over time, the course of therapy can be analyzed, and it can be illustrated whether interventions in patients suffering from obesity are successful [7,8].

There are many different methods for determining body composition, such as skinfold thickness measurement, hydrodensitometry, isotope dilution method, infrared reflection measurement, magnetic resonance imaging, computed tomography, or total body potassium counter [7]. Each method has advantages and disadvantages in terms of acquisition time, handling, and measurement accuracy. However, only a few methods are suitable for pediatric individuals, especially for children and adolescents with obesity. Moreover, the measurement of body composition of individuals with obesity is challenging. Hydrodensitometry is only applicable for children and adolescents who are able to submerge and hold their breath under water. Skinfold thickness measurement is not a suitable method for patients affected with obesity due to the enormous fat mass that the caliper cannot measure. Isotope dilution is expensive because of stable isotopes and analysis equipment. Neutron activation analysis and computed tomography (CT) are not suitable for children because of the radiation exposure. In contrast, quantitative magnetic resonance without ionizing radiation or dual-energy X-ray absorptiometry (DXA), which is the gold standard of the body composition methods [8,9], with minimal radiation exposure, is suitable for children but is often limited to research due to high costs [10,11,12,13,14].

Therefore, bioelectrical impedance analysis (BIA)-based methods are very practical, safe, easy to use, time-efficient, and cost-effective. Above all, they do not pose a burden for children and young adolescents and can also be used on children with obesity. So far, it has been shown that the BIA-based devices, TANITA that measures body composition in standing position and BIACORPUS that performs the composition analysis in supine position, are well suited for pediatric patients affected with obesity in clinical routine [15]. While earlier researcher showed poor agreement between each other [16], there is only little information available on BIA analysis for body composition compared to DXA in this specific vulnerable group of patients [17,18]. Limited and inconsistent data of BIA devices in pediatric patients with obesity have been published. Some studies revealed that BIA overestimated body fat percentage and fat mass and underestimated fat-free mass compared to DXA [19,20,21]. However, these results could not be confirmed by other studies, revealing that total body fat was underestimated using BIA compared to the gold standard [22,23]. The focus of previous research has been on individual BIA devices compared to DXA. Furthermore, many obesity outpatient clinics use BIA-based devices, and, in particular, TANITA and BIACORPUS are two very frequently used devices, and it would be very important to know whether TANITA or BIACORPUS compares better to the gold standard.

In recent years, air displacement plethysmography (ADP) has been increasingly used to measure the body composition of children [24,25,26], with a scientific focus especially on preterm infants [27,28]. In addition, data on air displacement plethysmography are available for adult obese patients [29,30], but comparative data of the ADP-based BODPOD device to clinically relevant body composition methods for a young obese population are missing. Therefore, it is of great interest to quantify the differences between the lying and standing BIA-based devices and ADP to the gold-standard method and to compare the methods with each other in children and adolescents suffering from obesity.

In our previous study, we focused on TANITA compared to BIACORPUS in a pediatric population affected by obesity. In a subgroup of seven patients, the body composition was measured with TANITA, BIACORPUS, BODPOD, and DXA [16]. However, based on this small sample size, no conclusions can be drawn. Hence, the next step was to investigate the four body composition devices in a larger sample size.

The aim of this study was to compare the two practical BIA devices, TANITA and BIACORPUS, which are commonly applied in clinical routine with the gold standard, DXA. We want to compare the lying and standing methods with the gold standard in young patients with obesity. The emphasis is to show whether TANITA or BIACORPUS is more accurate in measuring body composition and can therefore be recommended for regular examination of children and adolescent patients with obesity to assess treatment progress. In addition, BODPOD was compared with DXA to obtain data on this specific patient population. Finally, all methods were compared with each other to determine body fat percentage (BFP), fat mass (FM), and fat-free mass (FFM).

## 2. Materials and Methods

### 2.1. Study Participants

This study is a secondary analysis of the HIPstar trial that focused on the determination of the hip joint center location in overweight and obese individuals for clinical instrumented 3D gait analysis [31]. The study was approved by the Institutional Review Board of the Medical University of Vienna, Austria (EC Nr. 2060/2017). Written informed consent was obtained from all participants and legal representatives.

The inclusion criteria included children and adolescents aged from 10 to 18 years of both sexes and with a body mass index (BMI) above the 90th percentile, considered as overweight according to the central Europe BMI [32]. The BMI percentile curves are based on a reference population of children and adolescents aged 0–18 years. The BMI-for-age chart takes age, weight, height, and sex of the child into account [32]. Patients were recruited from the outpatient clinic of obesity, lipometabolic disorder, and nutritional medicine, Department of Pediatrics and Adolescent Medicine, Medical University of Vienna, from August 2020 to July 2022. The exclusion criteria were as follows: pregnancy, current syndrome associated with obesity (e.g., Prader–Willi syndrome), operations on upper/lower extremity within the last six months, neurological diseases, chronic joint diseases, and patients who were not able to walk barefoot.

### 2.2. Measurements

All measurements, both anthropometry and body composition measurements, were carried out according to standardized procedures and following the manufacturer’s instructions for each type of body composition device. Patients refrained from eating and drinking (e.g., sugar-sweetened beverages, tea, coffee, and alcohol) and vigorous physical activity for a minimum of 8 h prior examinations. Participants were measured with an empty bladder and while slightly dressed in underwear. The measurements were always carried out by the same trained personnel.

#### 2.2.1. Anthropometry

Anthropometric assessment included the body weight measured to the nearest 0.1 kg precision, and the body height was measured while the subject was barefoot and then rounded to the nearest 0.1 cm. Body mass index (BMI) was calculated by dividing weight in kilograms by the square of height (kg/m^2^). The following circumferences mid-upper arm, abdominal, hip, and waist circumference (all in cm) were measured. The waist-to-hip ratio was calculated by waist measurement (cm) divided by the hip circumference (cm), and the waist-to-height ratio was calculated by waist circumference (cm) divided by height (cm).

#### 2.2.2. Bioelectrical Impedance Analysis

The bioelectrical impedance analysis (BIA) measured the electrical properties of different body tissues. In order not to falsify the body water value, it was important that the patient’s bladder was empty before examination. In addition, it was clarified whether the patient did not exercise, eat, or drink anything prior to the body composition analysis. Moreover, the device was not used for individuals with metal allergy or pacemakers or any other kind of mechanical implants [33,34,35].

Two different devices, the TANITA (MC-980MA-N plus, Tokyo, Japan) and the BIACORPUS (RX 4000, Medical Health Care GmbH, Karlsruhe, Germany), were used, which are based on the bioelectrical impedance analysis technique.

To carry out the measurement with TANITA, the patient stood barefoot on the electrode platform of the scale with clean parallel positioned feet and fully extended knees. The patient was asked via the scale screen to take the two grip handles with the electrodes in their hands. The measurement was carried out with the body in standing position and straight arms down. The date of birth, gender, and body height were entered into the device. The TANITA was based on a multi-frequency 8-electrode system with a measurement frequency of 1 kHz, 5 kHz, 50 kHz, 250 kHz, 500 kHz, and 1000 kHz, and the impedance measurement ranged from 75.0 to 1,500.0 Ω, a measurement current of 90 µA or less, and an electric current range of 0.3 A. The device was used at room temperature, between 20 °C and 24 °C, which was within the temperature recommendations, and an age range from 5 to 99 years of age and a body height from 90.0 to 249.9 cm were appropriate to be measured [34].

With BIACORPUS (RX 4000, Medical Health Care GmbH, Karlsruhe, Germany), the patient received eight electrodes, two each on both hands and feet. The electrodes were connected to the device via cables. The patient was lying in supine position for approximately five minutes, and individual patients’ data of age, gender, body weight, and body height were entered into the device prior to the measurement. The patient received light electrical impulses, which were then transmitted at different speeds depending on the tissue [35]. A study by Potter et al. found that modern BIA systems, such as the InBody 770, demonstrated high precision, with a mean absolute error (MAE) of 3.4% for body fat percentage compared to DXA. These findings suggest that modern BIA devices can provide reliable estimates of body composition [36].

#### 2.2.3. Air Displacement Plethysmography

The air displacement plethysmography (ADP) utilized the relationship between pressure and volume to derive the body volume of the patient sitting in a chamber. This derivation of body volume, together with the measurement of body mass, allowed the calculation of body density and the subsequent estimation of the body fat percentage and fat-free mass [37]. The BODPOD (COSMED Inc., Chicago, IL, USA) was used within this study. The patient sat in a chamber in light underwear, and the head was covered with a bathing cap so that the protruding hair did not distort the measurement. In addition, earrings, eyeglasses, or similar items were not worn to avoid incorrect volume calculations. In accordance with the manufacturer, the device was not exposed to drafts to achieve optimal measurement results. Moreover, prior to the measurements, a calibration of the BODPOD was performed to ensure accurate body composition analysis outcomes [38,39].

#### 2.2.4. Dual-Energy X-Ray Absorptiometry

The dual-energy X-ray absorptiometry (DXA) measurement was performed with patients in the supine position, using DXA (Hologic Horizon system A, Hologic Inc., Marlborough, MA, USA) with the APEX system software version 5.6.0.5. With DXA, two X-ray sources are utilized with slightly different energy levels at the same time. This means that the different types of tissue can be distributed and compared between bone, muscles, and fat. The radiation exposure for patients of average body size and weight exposed at the age between 5 and 18 years and investigated with a Hologic Horizon should be in the range of 8.47–17.68 µSv [40]. This value was achieved in all corresponding patients in this study. The device used in this study was approved in accordance with national radiation protection legislation, and a daily quality assessment was carried out by certified radiology technicians. DXA of body composition is the gold standard of science and delivers, by far, the best results [41,42].

### 2.3. Statistical Analysis

Continuous variables were presented as means ± standard deviations and categorical variables were presented as frequencies (*n*) and percentiles (%). Each level of the repeated measures was tested for normality with the Kolmogorov–Smirnov test. The repeated measures analysis of variance (ANOVA) was used to determine the differences of body fat percentage, fat mass and fat-free mass between the four body composition methods. Compound symmetry, or sphericity, was verified by the Mauchly test. When the assumption of sphericity was not met, the significance of F ratios was adjusted according to the Greenhouse–Geisser when ε < 0.75 otherwise Huyn–Feldt correction was used. The repeated-measures ANOVA was conducted to compare body composition parameters × gender. In the case of significant results from the repeated-measures ANOVA, multiple Bonferroni-corrected pairwise comparisons were conducted to identify differences between the assessed methods. The Pearson correlation coefficients (r) were used to determine the correlation between body fat percentage, fat mass, and fat-free mass measured by the four different devices of body composition. The following strength-of-agreement categories were used: “very strong” was defined as having a correlation coefficient value of at least ±0.8, values between ±0.60 and ±0.79 suggested a “moderate–strong” correlation, values between ±0.30 and ±0.59 indicates a “fair” correlation, and values less than 0.3 determined a “poor” correlation [43,44]. Bland–Altman plots were created to determine the agreement between the different methods. The analysis indicated the mean difference ±1.96 SD and 95% limits of agreement [45,46,47]. This study used the SPSS statistics software (SPSS Inc., IBM Company, Chicago, IL, USA) version 26.0, and significance levels were set at a *p*-value < 0.05.

## 3. Results

### 3.1. Participants Characteristics

The study sample consisted of 32 patients, who met the inclusion criteria for the study. In total, 26 children and adolescents were finally measured with the different types of body composition devices. All children and adolescents were obese, with a BMI exceeding the 97th percentile [32]. An unpaired t-test did not indicate any significant differences (*p* < 0.05) in gender. However, male participants had an increased body weight, height, BMI, and abdominal and hip circumference compared to female participants. The participants’ characteristics are reported in Table 1.

### 3.2. Comparison of the Different Body Composition Methods

The results showed a statistically significant difference for body fat percentage, F(2.042, 48.999) = 9.921, *p* < 0.001, η_p_^2^ = 0.292, fat mass, F(2.983, 71.595) = 5.649, *p* = 0.002, η_p_^2^ = 0.191, as well as fat-free mass F(2.169, 52.060) = 7.491, *p* = 0.001, η_p_^2^ = 0.238. No significant gender differences were detected between the different methods (Table 2).

In detail, the comparison of the body composition measurements determined with TANITA, BIACORPUS, BODPOD, and DXA is shown in Table 3.

The repeated-measures ANOVA showed significant differences between BIACORPUS and DXA in regard to all three parameters. BIACORPUS underestimated body fat percentage (*p* < 0.001), as well as fat mass (*p* < 0.001), and it overestimated fat-free mass (*p* < 0.01) compared to DXA. TANITA underestimated BFP (*p* < 0.05) and FM (*p* < 0.001) in comparison to DXA. In contrast, BODPOD overestimated BFP (*p* < 0.05) and underestimated FFM (*p* < 0.05) compared to DXA.

The comparison of all methods with each other determined significant differences in all three parameters in BIACORPUS compared to BODPOD (BFP+FM+FFM: *p* < 0.001), and in TANITA versus BODPOD (BFP+FFM: *p* < 0.001; FM: *p* < 0.05). BIACORPUS underestimated body fat percentage (*p* < 0.05) and fat mass (*p* < 0.05) compared to TANITA.

### 3.3. Comparison of TANITA Versus DXA

**Figure 1:**
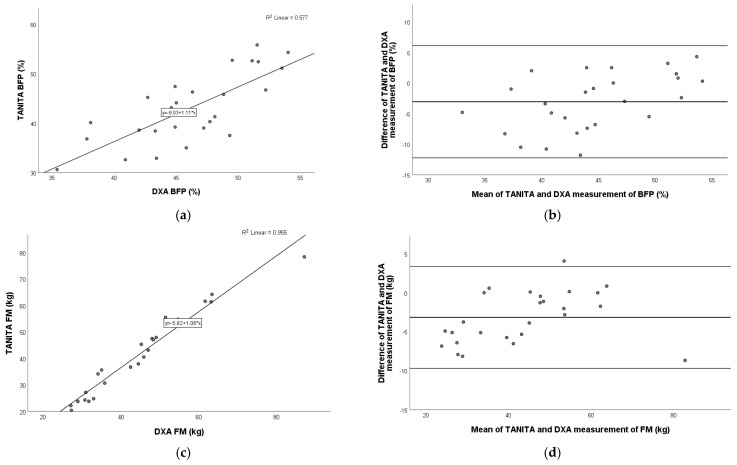

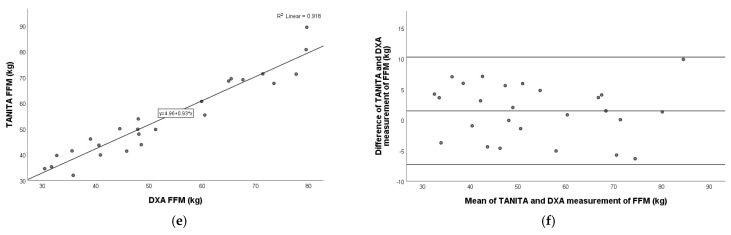
Pearson correlation analysis and Bland–Altman plot of TANITA vs. DXA in children with obesity. Left panel: The line represents the regression line. Pearson correlation between TANITA and DXA for BFP (**a**), FM (**c**), and FFM (**e**), with R^2^ and the equation of the best linear fit for each correlation plot. Right panel: The middle line indicates the mean difference, and the lower and upper lines represent the limits of agreement, from −1.96 to +1.96 SD. The middle region represents 95% CI. Bland–Altman plots obtained of TANITA and DXA for BFP (**b**), FM (**d**), and FFM (**f**). In comparison to DXA, TANITA underestimated BFP and FM and overestimated FFM. BFP, body fat percentage; FM, fat mass; FFM, fat-free mass.

A very strong positive relation was detected for fat mass (Pearson r = 0.977; *p* < 0.001), and fat-free mass (Pearson r = 0.958; *p* < 0.001), and a moderate–strong positive relation was shown for body fat percentage (Pearson r = 0.760; *p* < 0.001) between the TANITA and DXA devices. The Bland–Altman plot showed the following agreements for BFP (mean: −3.1%; 95% limits of agreement: −12.2% to 6.1%), FM (mean: −3.2 kg; 95% limits of agreement: −9.7 kg to 3.4 kg), and FFM (mean: 1.5 kg; 95% limits of agreement: −7.3 kg to 10.3 kg). These relationships are shown graphically in the following figure (Figure 1).

### 3.4. Comparison of BIACORPUS Versus DXA

**Figure 2:**
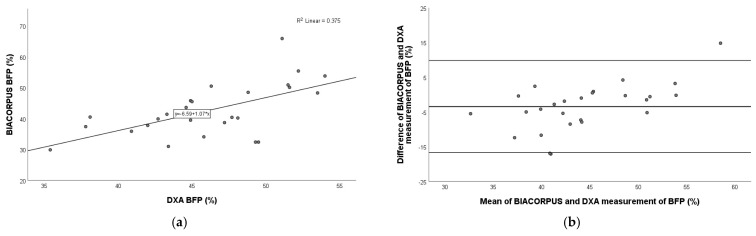

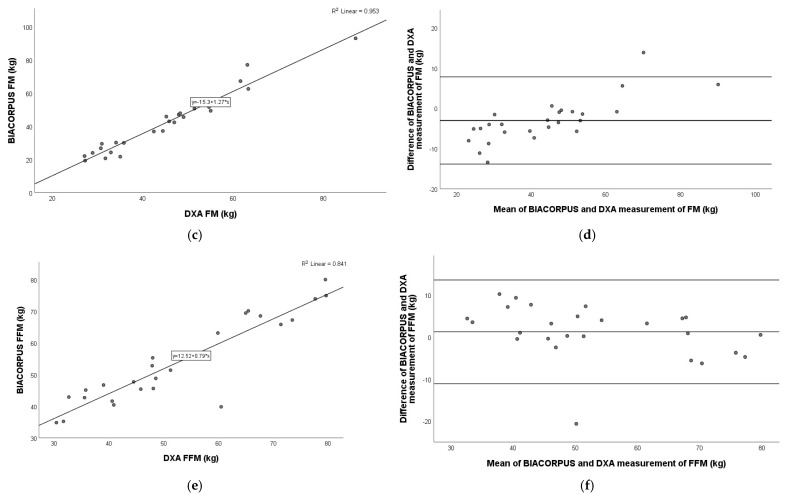
Pearson correlation analysis and Bland–Altman plot of BIACORPUS vs. DXA in children with obesity. Left panel: The line represents the regression line. Pearson correlation between BIACORPUS and DXA for BFP (**a**), FM (**c**), and FFM (**e**), with R^2^ and the equation of the best linear fit for each correlation plot. Right panel: The middle line indicates the mean difference, and the lower and upper lines represent the limits of agreement, from −1.96 to +1.96 SD. The middle region represents 95% CI. Bland–Altman plots obtained of BIACORPUS and DXA for BFP (**b**), FM (**d**), and FFM (**f**). In comparison to DXA, BIACORPUS underestimated BFP and FM and overestimated FFM. BFP, body fat percentage; FM, fat mass; FFM, fat-free mass.

As shown in Figure 2, a very strong positive relation was observed for fat mass (Pearson r = 0.976; *p* < 0.001) and fat-free mass (Pearson r = 0.917; *p* < 0.001), and a moderate–strong relation was observed for body fat percentage (Pearson r = 0.612; *p* = 0.001) between BIACORPUS and DXA.

The Bland–Altman plot showed the best agreement with FFM (mean: 1.3 kg; 95% limits of agreement: −11.1 kg to 13.6 kg), followed by BFP (mean: −3.5%; 95% limits of agreement: −16.7% to 9.8%), and FM (mean: −3.0 kg; 95% limits of agreement: −13.9 kg to 7.8 kg) (Figure 2).

### 3.5. Comparison of BODPOD Versus DXA

Our results showed a very strong, significant positive correlation (*p* < 0.001) in all three parameters measured with the BODPOD and DXA devices: BFP (Pearson r = 0.866), FM (Pearson r = 0.952), and FFM (Pearson r = 0.976).

**Figure 3:**
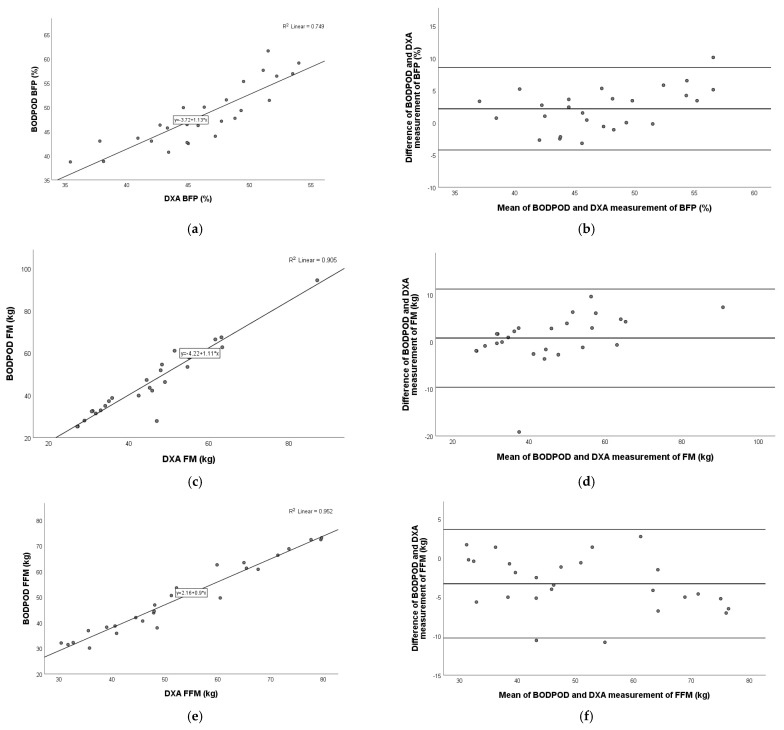
Pearson correlation analysis and Bland–Altman plot of BODPOD vs. DXA in children with obesity. Left panel: The line represents the regression line. Pearson correlation between BODPOD and DXA for BFP (**a**), FM (**c**), and FFM (**e**), with R^2^ and the equation of the best linear fit for each correlation plot. Right panel: The middle line indicates the mean difference, and the lower and upper lines represent the limits of agreement, from −1.96 to +1.96 SD. The middle region represents 95% CI. Bland–Altman plots obtained of BODPOD and DXA for BFP (**b**), FM (**d**), and FFM (**f**). In comparison to DXA, BODPOD underestimated BFP and FM and overestimated FFM. BFP, body fat percentage; FM, fat mass; FFM, fat-free mass.

The Bland–Altman plot showed the best agreement with FM (mean: 0.8 kg; 95% limits of agreement: −9.6 kg to 11.2 kg), followed by BFP (mean: 2.1%; 95% limits of agreement: −4.2% to 8.5%) and FFM (mean: −3.3 kg; 95% limits of agreement: −10.3 kg to 3.6 kg) (Figure 3).

### 3.6. Comparison of TANITA Versus BIACORPUS

**Figure 4:**
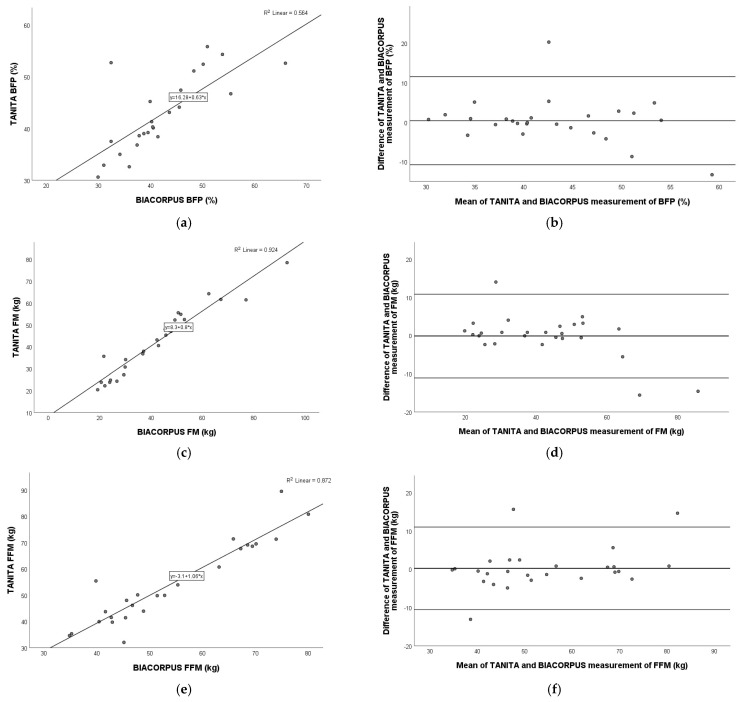
Pearson correlation analysis and Bland–Altman plot of TANITA vs. BIACORPUS in children with obesity. Left panel: The line represents the regression line. Pearson correlation between TANITA and BIACORPUS for BFP (**a**), FM (**c**), and FFM (**e**), with R^2^ and the equation of the best linear fit for each correlation plot. Right panel: The middle line indicates the mean difference, and the lower and upper lines represent the limits of agreement, from −1.96 to +1.96 SD. The middle region represents 95% CI. Bland–Altman plots obtained of TANITA and BIACORPUS for BFP (**b**), FM (**d**), and FFM (**f**). In comparison to BIACORPUS, TANITA overestimated BFP and FM and underestimated FFM. BFP, body fat percentage; FM, fat mass; FFM, fat-free mass.

Figure 4 shows a very strong positive correlation for fat mass (Pearson r = 0.962; *p* < 0.001) and fat-free mass (Pearson r = 0.934; *p* < 0.001) and a moderate–strong correlation for body fat percentage (Pearson r = 0.751; *p* < 0.001) between TANITA and BIACORPUS. Figure 4 represents the Bland–Altman plot and the agreement for BFP (mean: 0.4%; 95% limits of agreement: −10.8% to 11.5%), FM (mean: −0.1 kg; 95% limits of agreement: −11.1 kg to 10.8 kg), and FFM (mean: −0.2 kg; 95% limits of agreement: −10.6 kg to 11.0 kg).

### 3.7. Comparison of TANITA Versus BODPOD

A very strong, significant positive correlation was revealed in all three variables, each with a *p*-value of < 0.001 for BFP (Pearson r = 0.835), FM (Pearson r = 0.940), and FFM (Pearson r = 0.964) between TANITA and BODPOD.

**Figure 5:**
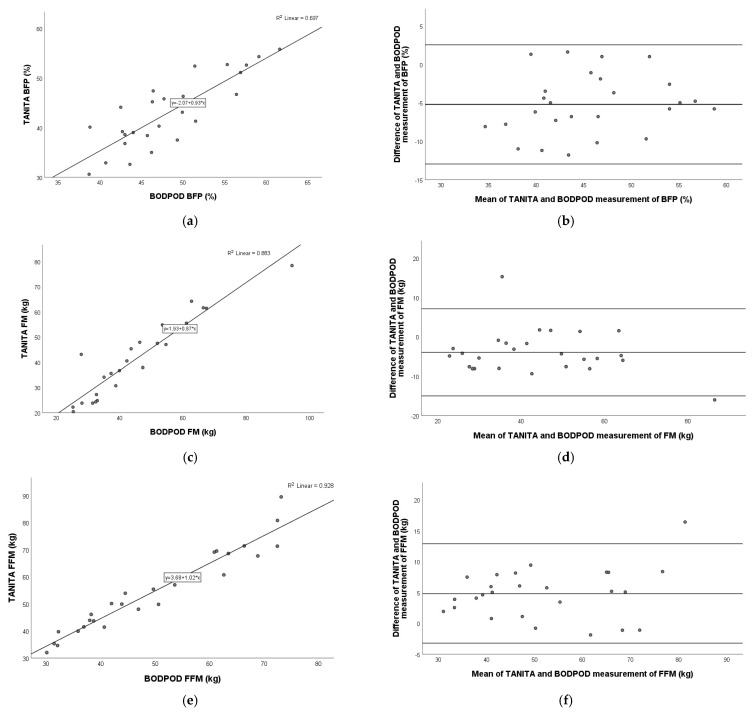
Pearson correlation analysis and Bland–Altman plot of TANITA vs. BODPOD in children with obesity. Left panel: The line represents the regression line. Pearson correlation between TANITA and BODPOD for BFP (**a**), FM (**c**), and FFM (**e**), with R^2^ and the equation of the best linear fit for each correlation plot. Right panel: The middle line indicates the mean difference, and the lower and upper lines represent the limits of agreement, from −1.96 to +1.96 SD. The middle region represents 95% CI. Bland–Altman plots obtained of TANITA and BODPOD for BFP (**b**), FM (**d**), and FFM (**f**). In comparison to BODPOD, TANITA underestimated BFP and FM and overestimated FFM. BFP, body fat percentage; FM, fat mass; FFM, fat-free mass.

The Bland–Altman plot showed the following agreements for BFP (mean: −5.2%; limits of agreement: −13.0% to 2.5%), FM (mean: −4.0 kg; 95% limits of agreement: −15.0 kg to 7.1 kg), and FFM (mean: 4.8 kg; 95% limits of agreement: −3.2 kg to 12.8 kg) (Figure 5).

### 3.8. Comparison of BIACORPUS Versus BODPOD

A very strong positive correlation was observed in the comparison of BIACORPUS and BODPOD for fat mass (Pearson r = 0.940; *p* < 0.001), fat-free mass (Pearson r = 0.933; *p* < 0.001), and a moderate–strong correlation for body fat percentage (Pearson r = 0.661; *p* < 0.001).

**Figure 6:**
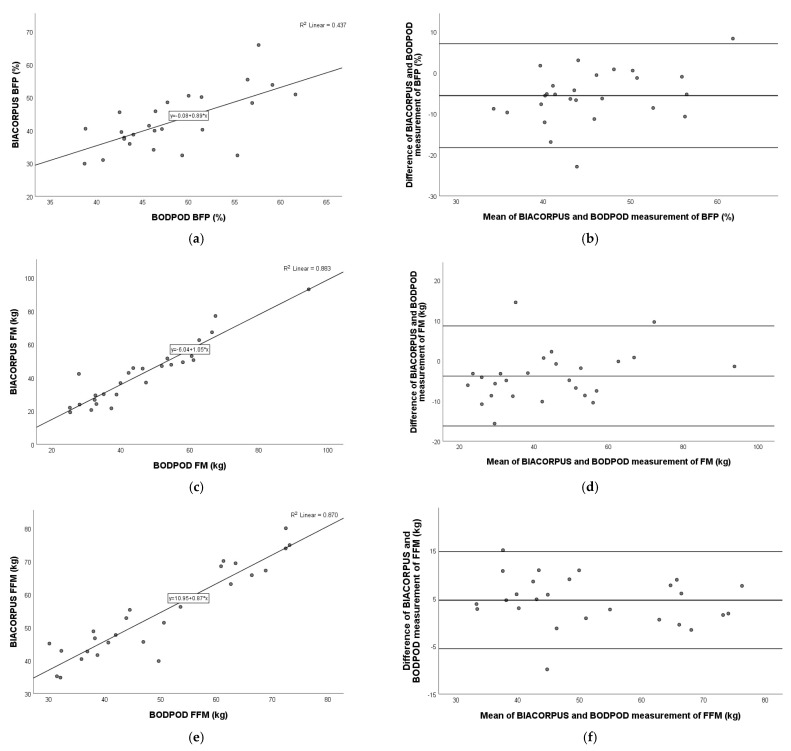
Pearson correlation analysis and Bland–Altman plot of BIACORPUS vs. BODPOD in children with obesity. Left panel: The line represents the regression line. Pearson correlation between BIACORPUS and BODPOD for BFP (**a**), FM (**c**), and FFM (**e**), with R^2^ and the equation of the best linear fit for each correlation plot. Right panel: The middle line indicates the mean difference, and the lower and upper lines represent the limits of agreement, from −1.96 to +1.96 SD. The middle region represents 95% CI. Bland–Altman plots obtained for BIACORPUS and BODPOD for BFP (**b**), FM (**d**), and FFM (**f**). In comparison to BODPOD, BIACORPUS underestimated BFP and FM and overestimated FFM. BFP, body fat percentage; FM, fat mass; FFM, fat-free mass.

The Bland–Altman plot showed the best agreement with FM (mean: −3.8 kg; 95% limits of agreement: −16.2 kg to 8.6 kg), followed by FFM (mean: 4.6 kg; 95% limits of agreement: −5.5 kg to 14.7 kg) and BFP (mean −5.6%; 95% limits of agreement: 18.3% to 7.1%) (Figure 6).

## 4. Discussion

The present study analyzed and compared four different methods of body composition analysis in young patients with obesity. The focus was on practically usable methods, TANITA and BIACORPUS, compared to the gold standard, DXA. Comparison with the BODPOD was also used to obtain data on this specific patient population. In addition, all body composition devices were compared to each other.

Our results highlighted that all three more practical and cost-efficient methods, TANITA, BIACORPUS, and BODPOD, have shown differences from the gold standard. The BODPOD overestimated body fat percentage and fat mass, and it underestimated fat-free mass compared to DXA. The exact opposite was observed with the two BIA devices. TANITA and BIACORPUS underestimated the BFP and FM and overestimated the FFM. The comparison of all methods to each other detected little agreement for body fat percentage, fat mass, and fat-free mass with the Bland–Altman plots. However, the non-invasive and user-friendly TANITA scale was the BIA method, which was most comparable to DXA. The Bland–Altman plots demonstrated better agreement in BFP with TANITA than in BIACORPUS, compared to DXA. Moreover, the mean values did not exceed the clinically relevant cut-off levels of ±5% for body fat percentage, ±5 kg for fat mass, and ±5 kg fat-free mass [15]. Therefore, TANITA can be utilized to determine the body composition and to evaluate follow-up visits and therapy progress in children and adolescents with obesity.

However, the limitations of the different body composition methods must be mentioned. BIA-based devices like TANITA and BIACORPUS are non-invasive methods but have their limitations, especially in terms of hydration, body type, health conditions, food and beverage intake, physical activity, fluid retention, blood volume, placement and number of electrodes, environmental factors, and inconsistencies across different BIA models. The accuracy and precision of BIA devices can vary when factors are outside the average-based model. Dehydration might result in an overestimation of total body fat, and overhydration of the opposite. Physical activity, as well as food and beverage intake, directly before the measurement can affect fluid intake and blood flow. Kidney diseases, diabetes, edema, or other fluid retention in the body might show inaccurate results. Extreme body types and shapes in terms of muscle mass or total body fat might lead to unreliable results. Moreover, TANITA and BIA do not provide detailed analysis of specific areas of the body like DXA does. Room temperature, humidity, or altitude can affect the body’s conductivity and thus possibly distort the results. The proper placement and contact of electrodes, as well as the number of electrodes, might affect the results, as BIA devices based on multi-frequency eight-electrode systems show technical advantages compared to single-frequency four-electrode systems [48,49,50,51,52]. The BODPOD is expensive and therefore often restricted to specialized clinics and research facilities, and the measurement is time-consuming. The accuracy of this device is dependent on calibration and maintenance; it is sensitive to temperature, air, and humidity changes outside and inside the chamber; and during measurements, children, in particular, should not move, and they should breathe regularly. The accuracy might be limited in children under the age of 6 years and or individuals with very low weight or small body volume [24,53]. DXA is widely considered to be one of the most accurate methods for measuring body composition, but these limitations should be considered when choosing this method. DXA devices are expensive und usually only available in specialized medical facilities. For DXA scans, trained professionals are needed to ensure proper positioning and accurate results. In comparison to BIA devices, DXA scans can take longer, particularly to determine the multiple regions of the body. Although the radiation dose used in DXA is very low, it still involves exposure to X-rays and should therefore not be used frequently in short intervals, especially in vulnerable collectives, such as children and adolescents. Even though DXA is a sophisticated method and can measure bone density, in addition to fat mass and fat-free mass, it cannot measure the distribution of fat across the body as precisely as other techniques, like MRI or CT [54,55,56].

BODPOD seems to show high precision in regard to body composition measurement in normal-weight adults [57] and children [32]. However, we detected significant differences between BODPOD and DXA in our young group affected with obesity. Our results confirm the findings of Ball and Altena, who showed that the differences between BODPOD and DXA increased with body fat percentage [33]. Another reason for the differences could be the density of the fat-free mass, which is lower in children with obesity compared to children of normal weight [58]. The measurement of FM and FFM using BODPOD assumes a constant FFM density according to gender and age [59]. Therefore, this should be adapted for the pediatric population with obesity to decrease the bias in measurements with ADP devices [60]. In contrast, DXA has the ability to determine the different FFM densities and to distinguish between lean mass and bone mineral content [55].

The results of this study revealed that both methods based on bioimpedance analysis statistically differed. The TANITA scale measured body fat percentage and fat mass higher than that measured by BIACORPUS. Nevertheless, the fat-free mass was significantly higher when determined by BIACORPUS. These results are consistent with those of previous studies [15,16], showing the importance of not using devices interchangeably for body composition analysis. The differences between the two BIA methods might be explained by the different body positions of the patients during the body composition measurement. The sitting and lying positions affect the body fluid distribution and have an impact on reactance and resistance, and thus on intracellular and extracellular water [61,62]. Nevertheless, no impact on fat mass and fat-free mass was determined [62]. Hamilton-James et al. ascertained that the standing BIA method is reliable and has a high within-method precision. Additionally, this high level of precision was also observed with the supine-positioned BIA technique, but the two methods should not be used interchangeably [63]. Our results are consistent with those of previous studies showing that the BIA measurements in supine and standing position differ from each other [63]. It is important to note that some studies used different BIA models. For example, Parker et al. used a “foot-to-foot” device [64]. The difference between “foot-to-foot” bioimpedance analyses, in which the electrodes are only placed on the feet, and “hand-to-foot” bioimpedance analyses, in which the examiners also have electrodes on their hands, has not yet been well studied. Long et al. examined the difference in body fat percentage between the BIA models, “foot to foot,” “hand to foot,” and “hand to hand” [65]. These measurements were then compared to BODPOD and to the anthropometric measurements. This study indicated that all examined BIA models provided similar results to the BODPOD. However, with increased hip circumference or waist circumference, the difference between the methods increased [65]. In our study sample, an increased waist, as well as hip, circumference was observed, especially in male individuals, and might have caused these differences between the methods. Interestingly, another study revealed that the underestimation of fat percentage in overweight and obese adolescents was less pronounced using the “foot-to-foot” method in standing position than using the “hand-to-foot” method in supine position [66]. A possible reason might be the body position during BIA analysis. In the lying position, the body fluids are more evenly distributed. In contrast, gravity affects the distribution of body fluids with an increased concentration in the lower extremities in the standing position and might affect the measurement [63,67,68]. The results of these studies suggest that it is important to compare not only the bioimpedance analysis with the gold-standard methods, such as the BODPOD or the DXA scan, but also the BIA models with each other, especially in patients with obesity.

The manufacturers do not provide any information on precision and accuracy, especially not for the different models. In children and adolescents with obesity, data on the precision and accuracy of BIA devices are limited [20,21,51,69,70]. The results of published studies are very inconsistent because the results depend on which method and body composition model was used, which methods were compared, and in which study sample the devices were analyzed. The comparison of both BIA methods showed that the results of TANITA were more similar to those of the gold-standard method, DXA. Kabiri et al. demonstrated excellent test–retest reliability in TANITA compared to DXA in primary-school children. Moreover, the analysis of body fat percentage revealed high specificity in a subgroup of children with obesity [18]. In addition, similar results were detected in a small group of adolescents with obesity [17]. However, it must be mentioned that a different TANITA model was used. According to the literature, BIA results are valid in healthy individuals with a BMI up to 34 kg/m^2^ [40], and in our patients, 62% had a BMI exceeding this cut-off (BMI range: 35–52 kg/m^2^).

TANITA is easier to handle and faster than the other measuring methods. In addition, measuring with TANITA during a routine examination in the outpatient clinic is possible without much effort; the device requires little space, is more cost-effective, and is suitable for very young patients. Moreover, if you want to carry out several measurements on a patient in order to assess therapy progress, bioimpedance analysis with the TANITA scale is recommended. Measuring with BIACORPUS takes more time compared to TANITA. It should be noted that both DXA and BODPOD measurements are more complex, time-consuming, and expensive, and therefore follow-up examinations cannot be carried out as frequently in clinical routine. In addition, these devices are more often used within studies. However, follow-up visits are essential to better assess the alterations of weight loss or weight gain, particularly for children and adolescents with obesity. This study showed that the four methods differed significantly, and therefore, it would be best to choose one body composition device and to use it continuously for follow-ups to prevent any bias.

This study has some limitations that need to be recognized. In both BIA methods, in lying and standing position, the legs should not touch each other. The problem was that our patients have a very large thigh circumference, so that the inner thighs would touch each other. BIA analyzes the body composition by sending a small electrical current through the legs, arms, and trunk. The contact between the extremities might affect the impedance and distort the results. It is therefore questionable to what extent the non-ideal body position influenced the measurement of the body composition results. The group of boys was overrepresented, which must be taken into account when looking at the results.

The major strength of this study was that all body composition examinations were carried out on patients under standardized conditions by the same trained staff members to prevent any measurement errors. Another strength is that the four body composition measurements were always carried out on the same day for each patient to ensure consistency and accuracy, and to provide reliable and comparable data. Both BIA devices use electrodes on the hands and feet and are based on eight-electrode multi-frequency devices, and they can therefore be compared with each other.

## 5. Conclusions

In particular, all three more practical and cost-efficient methods have shown differences from dual-energy X-ray absorptiometry. In addition, the comparison of all methods with each other indicates that the devices should not be used alternately to measure the body composition of a young person with obesity. BIA should not be used interchangeably with DXA in clinical settings. A measurement with DXA is recommended to determine the precise body composition, especially body fat. Otherwise, when taking into account practicability and use in everyday clinical practice TANITA, even though it showed differences in DXA, can also be used, which was the most comparable BIA method to the gold standard. Thus, we recommend using TANITA to determine the individual body composition in young patients with obesity to assess therapy progress in clinical routine. When using BODPOD as an alternative to DXA, caution is warranted since we found relevant differences between both methods.

## Figures and Tables

**Table 1:** Patients’ demographic data.

Characteristics	Patients (*n* = 26)	Male (*n* = 17)	Female (*n* = 9)
Age (years)	13.1 ± 2.5	13.2 ± 2.2	12.9 ± 3.0
Height (cm)	162.7 ± 10.5	165.4 ± 10.7	158.8 ± 9.8
Weight (kg)	95.8 ± 27.6	101.5 ± 30.1	87.6 ± 21.4
BMI (kg/m^2^)	35.6 ± 6.9	36.4 ± 7.2	34.4 ± 6.7
Mid-upper arm circumference (cm)	35.0 ± 4.4	35.0 ± 4.3	35.2 ± 4.9
Abdominal circumference (cm)	103.7 ± 12.4	109.0 ± 12.1	97.2 ± 9.8
Hip circumference (cm)	112.3 ± 15.8	116.8 ± 15.3	106.6 ± 15.6
Waist circumference (cm)	114.8 ± 20.1	115.4 ± 22.7	114.6 ± 15.9
Waist-to-hip ratio	0.93 ± 0.20	0.98 ± 0.24	0.85 ± 0.06
Waist-to-height ratio	0.64 ± 0.06	0.66 ± 0.06	0.61 ± 0.06

Results are presented as mean ± SD or as number (%). SD, standard deviation; BMI, body mass index.

**Table 2:** Outcomes of repeated-measures ANOVA for BFP, FM, FFM, and gender.

	ANOVA			
	F	Df	*p*	η_p_^2^
**BFP (%)**				
BFP	9.921	2.042	0.000	0.292
Gender	1.905	1	0.180	0.074
BFP × gender	2.929	2.042	0.062	0.109
**FM (kg)**				
FM	5.649	2.983	0.002	0.191
Gender	0.168	1	0.686	0.007
FM × gender	1.091	2.983	0.358	0.043
**FFM (kg)**				
FFM	7.491	2.169	0.001	0.238
Gender	3.424	1	0.077	0.125
FFM × gender	3.032	2.169	0.053	0.112

BFP, body fat percentage; FM, fat mass; FFM, fat-free mass.

**Table 3:** Comparison of TANITA, BIACORPUS, BODPOD, and DXA measurement.

Parameter	TANITA	BIACORPUS	BODPOD	DXA
BFP (%)	43.0 ± 7.1	39.9 ± 8.4	48.5 ± 6.1	46.4 ± 4.6
FM (kg)	41.7 ± 15.3	39.0 ± 17.0	45.9 ± 16.5	45.1 ± 14.2
FFM (kg)	53.7 ± 15.0	56.3 ± 14.6	48.7 ± 13.8	51.8 ± 15.0

Results are presented as mean ± SD. DXA, dual-energy X-ray absorptiometry; BFP, body fat percentage; FM, fat mass; FFM, fat-free mass.

## Data Availability

Data that support the findings of this study are available upon reasonable request to the corresponding author.
